# Computational Simulation Expands Understanding of Electrotransfer-Based Gene Augmentation for Enhancement of Neural Interfaces

**DOI:** 10.3389/fnins.2019.00691

**Published:** 2019-08-06

**Authors:** Amr Al Abed, Jeremy L. Pinyon, Evelyn Foster, Frederik Crous, Gary J. Cowin, Gary D. Housley, Nigel H. Lovell

**Affiliations:** ^1^Graduate School of Biomedical Engineering, University of New South Wales, Sydney, NSW, Australia; ^2^Translational Neuroscience Facility, Department of Physiology, School of Medical Sciences, University of New South Wales, Sydney, NSW, Australia; ^3^National Imaging Facility, Centre for Advanced Imaging, University of Queensland, Brisbane, QLD, Australia

**Keywords:** electroporation, electric field, cochlea, computational modeling, field mapping, electrode impedance

## Abstract

The neural interface is a critical factor in governing efficient and safe charge transfer between a stimulating electrode and biological tissue. The interface plays a crucial role in the efficacy of electric stimulation in chronic implants and both electromechanical properties and biological properties shape this. In the case of cochlear implants, it has long been recognized that neurotrophins can stimulate growth of the target auditory nerve fibers into a favorable apposition with the electrode array, and recently such arrays have been re-purposed to enable electrotransfer (electroporation)-based neurotrophin gene augmentation to improve the “bionic ear.” For both this acute bionic array-directed electroporation and for chronic conventional cochlear implant arrays, the electric fields generated in target tissue during pulse delivery are central to efficacy, but are challenging to map. We present a computational model for predicting electric fields generated by array-driven DNA electrotransfer in the cochlea. The anatomically realistic model geometry was reconstructed from magnetic resonance images of the guinea pig cochlea and an eight-channel electrode array embedded within this geometry. The model incorporates a description of both Faradaic and non-Faradaic mechanisms occurring at the electrode-electrolyte interface with frequency dependency optimized to match experimental impedance spectrometry measurements. Our simulations predict that a tandem electrode configuration with four ganged cathodes and four ganged anodes produces three to fourfold larger area in target tissue where the electric field is within the range for successful gene transfer compared to an alternate paired anode-cathode electrode configuration. These findings matched *in vivo* transfection efficacy of a green fluorescent protein (GFP) reporter following array-driven electrotransfer of the reporter-encoding plasmid DNA. This confirms utility of the developed model as a tool to optimize the safety and efficacy of electrotransfer protocols for delivery of neurotrophin growth factors, with the ultimate aim of using gene augmentation approaches to improve the characteristics of the electrode-neural interfaces in chronically implanted neurostimulation devices.

## Introduction

The neural interface can be considered as the predominant factor limiting further advancement of chronic neurostimulation technologies such as the cochlear implant. Deterioration of the neural interface, due to surgical implant procedure, fibrosis, and strong foreign body responses among other reasons, leads to poor coupling of implanted multi-electrode arrays to target neurons. In the case of cochlear implants, inadequate neural selectivity due to high excitation thresholds and resulting current spread prevents the improvement of positional recruitment – based pitch perception and speech perception outcomes in implant recipients (O’Leary et al., 2009).

Attempts to improve the cochlear implant neural interface have included delivery of neurotrophic factors through a variety of approaches to regenerate nerve fibers, reducing the distance between target neuron processes and the electrode array (Pettingill et al., 2008; Richardson et al., 2009; Wise et al., 2010; Atkinson et al., 2012). Within this context, close field electroporation (CFE) has shown promising potential for the electrotransfer of gene constructs to cells within localized tissue region. Because CFE utilizes the cochlear implant electrode array itself to drive transfection with highly specific localization proximal to the array, CFE is able to achieve meaningful tropism in the context of enhancement of the bionic ear interface (Pinyon et al., 2014). This potentially provides superiority to alternate approaches such as those that are cell-based, viral-based or through direct protein infusion, which do not provide cochlear implant-guided tropic support. An effect that was successfully demonstrated by transfecting localized regions of mesenchymal cells within the cochlea surrounding the electrode array. When these cells produce recombinant neurotrophic factors, a concentration gradient is established, directing regrowth of the cochlear neuron peripheral processes directly toward the transfected mesenchymal cells and thus the electrodes of the cochlear implant array. Furthermore, changing the configuration of active and return electrodes used for gene electrotransfer in an eight-channel array can affect the number of cells transfected both *in vitro* using cultured human embryonic kidney-293 (HEK293) cells and *in vivo* in the guinea pig cochlea (Pinyon et al., 2014; Browne et al., 2016).

It is hypothesized that successful electrotransfer of gene constructs is correlated to the strength of electric fields generated by the electroporation pulses in target tissue (Browne et al., 2016). While electric field mapping has been carried out *in vitro* (Browne et al., 2016), electric field density and the resulting transfection patterns produced within the cochlea are not well understood due to the difficulty of mapping electric fields in a complex geometry such as the cochlea. Knowledge of optimal stimulation parameters for targeted gene electrotransfer is needed to achieve high efficiency and enable spatial control of the transfected region.

To address this knowledge gap, in this study we develop an anatomically realistic computational model of the guinea pig cochlea to enable simulation of electroporation pulses produced by array-driven stimulation. We hypothesize that the electric fields predicted by our computational model are correlated to transfection localization and density observed experimentally. The validated model can then be utilized to design electrode configurations for CFE that optimize acute targeted gene electrotransfer in the cochlea, facilitating broad improvement of chronically implanted neural interfaces in the cochlea.

## Materials and Methods

### Electrode Impedance Characterization

Electrochemical impedance spectrometry (EIS) was conducted on an eight channel Pt electrode array (part no. Z60276; Cochlea, Australia). The array consisted of eight platinum electrodes with diameter 350 μm, length 300 μm, and spacing of 300 μm.

The electrode array was immersed in 40 mL of Dulbecco’s phosphate-buffered saline (DPBS) and measurements were conducted using an EA 163 three electrode potentiostat (eDAQ, Australia). The collecting/return electrode was chosen to be a piece of platinum scrap with dimensions significantly larger than the cochlea working electrodes. The reference electrode was a Ag/AgCl electrode. To ensure that distances between electrodes remained constant, all three electrodes ran into the solution parallel to the axis of the beaker. The cochlea electrode array and return and reference electrodes were vertically aligned and submerged to approximately the mid-level of the solution. Electrodes were positioned so that the reference electrode was in the midline between the working electrode (cochlear electrode array) and the return electrode. The electrode system was placed within a Faraday cage to minimize electrical interference.

The ERZ100 software (eDAQ, Australia) provided the functionality to perform the EIS measurements. It contains a function generator which provided the stimulation waveform as well as a frequency response analyzer that determined impedance magnitude and phase angle. Each of the eight electrodes on the cochlea array was analyzed individually, with order being randomized. For each electrode, frequency was swept in the 0.1–10 kHz range with AC amplitude of 30 mV.

### *In vivo* Electroporation

All experimental procedures were conducted in accordance with the NSW Animal Research Act 1985, NSW Animal Research Regulations 2010, and Australian Code for the Care and Use of Animals for Scientific Purposes 8th Edition 2013, and were approved by the University of New South Wales Animal Care and Ethics Committee (ACEC approval number 10/81A).

The data on cochlear mesenchymal cell transfection with green fluorescence protein reporter (GFP) expression – encoding plasmid DNA used in this study to validate the cochlear implant array computational modeling was derived from the initial report of close-field electroporation (Pinyon et al., 2014). The methodology is further expanded in Browne et al. (2016) and Pinyon et al. (2019). Briefly, colored guinea pigs of both sexes 300–900 g in weight were anesthetized using isoflurane. GFP reporter plasmid DNA incorporating a cytomegalovirus promoter [2 μg/μl in Tris (hydroxymethyl)aminomethane (Tris) buffered saline] was delivered to the cochlea via the round window at a rate of 20 μl per 40 s using a Narishige IM-1 microdrive pump (Narishige, Japan). A pre-clinical research cochlear implant electrode array (part no. Z60276; Cochlea, Australia) was then inserted into the basal turn of the scala tympani via the round window. Prior to electroporation the total impedance of the system was determined using the resistance-measuring mode of a CUY21 square wave electroporator (Nepa Gene, Japan). For electroporation, constant voltage pulses were delivered via the electrode array in either of two electrode configurations: “alternate” whereby alternating electrodes in the array were set as anode and cathode (*n* = 4), or “tandem” whereby four neighboring electrodes were set as the anode and the next four as cathode (*n* = 7). In selected experiments, as a no electrotransfer, the array was inserted but the electroporation pulse train was not applied (*n* = 2). The cochlear implant array was removed within 5 min of the DNA electrotransfer and the surgical field was closed. The guinea pigs were euthanized after 3–4 days and following fixation with 4% paraformaldehyde (PFA), the cochlea was removed and decalcified in 8% ethylenediaminetetraacetic acid (EDTA) in 0.1 M sodium phosphate buffer at 4°C for 2–3 weeks. The nuclear-localized GFP reporter signal in the target mesenchymal cell area adjacent to the electrode array was visualized using a NLO710 confocal laser scanning microscope (Zeiss, Germany). Fluorescence was excited at 488 nm using an argon laser and emissions collected at 492–548 nm wavelengths.

### MRI and Image Reconstruction

**FIGURE 1:**
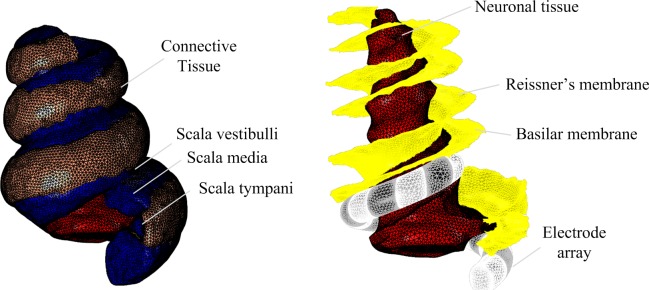
3D reconstruction of guinea pig cochlea.

For high resolution magnetic resonance imaging (MRI) to delineate the spatial features of the cochlea, a 500 g male guinea pig was euthanized via intraperitoneal injection of pentobarbital and then cardiac perfused using 50 ml of saline containing 0.5% sodium nitroprusside, followed by fixation with 100 ml of phosphate buffered 4% PFA. The cochleae were removed and further fixed overnight at 4°C. Magnetic resonance images of the guinea pig cochlea were obtained using a 16.4T AV700 MRI system at the National Imaging Facility, Australia, with a 12.5 μm × 12.9 μm × 12.5 μm spatial resolution, and averaging of four repeats and the following image sequence parameters: 3D gradient echo, Fast Low Angle Shot (FLASH), TR = 40, TE = 5.5, pulse = 35°. The total acquisition time was 22.5 h. The soft tissue, cochlear canals and nerve tissue were segmented and 3D masks reconstructed in Mimics v19.0 (Materialise, Belgium) using standard thresholding and morphological filters as well as tracing tools (Figure 1). Further smoothing and morphological filtering was conducted to define the Reissner’s membrane boundary (between scala vestibuli and scala media) and the basilar membrane boundary (between scala media and scala tympani).

The geometry for the electrode array was created in SolidWorks 2016 (Dassault Systèmes, France) as a spiral with variable radius and uniform pitch. The curvature was defined based on anatomical measurements to ensure that the electrode array would fit within the scala tympani (Figure 1). The positioning within the scala tympani structure was achieved by manual rotation and translation in 3-Matic v11.0 (Materialise, Belgium). Meshes for anatomical structures and the electrode array were generated, smoothed and assembled in 3-Matic v11.0 (Materialise, Belgium) before export to COMSOL Multiphysics v5.3a (COMSOL AB, Sweden) for further refinement.

### Finite Element Modeling

A computational model was built to simulate array-driven electroporation in the reconstructed cochlea using the finite-element solver software COMSOL Multiphysics.

The cochlea geometry with electrode array embedded was meshed using tetrahedral volumetric elements (1,368,698) and triangular boundary elements (125,799). The average element skewness was 0.67, on a scale from 0 to 1 with 1 being a perfect equilateral tetrahedron or triangle.

The cochlea was assumed to be electrically passive, with each anatomical structure having an isotropic specific conductivity σ (S.m^-1^) (Table 1 for values) with the extracellular voltage distribution V (V) governed by Poisson’s equation:

∇⋅(−σ∇V)=0

The basilar membrane and Reissner’s membranes were modeled as boundaries assigned with numerical thicknesses (d_s_) of 90 μm and 20 μm, respectively, representing contact impedances:

$$\hat{n}.\vec{J_{1}}=\frac{\sigma }{d_{s}}(V_{1}-V_{2})$$

$$\hat{n}.\vec{J_{2}}=\frac{\sigma }{d_{s}}(V_{2}-V_{1})$$

where $\hat{n}$ is the unit vector normal to the boundary, V_1_ and V_2_ are the electric potentials on either side of the boundary, and $\vec{J_{1}}$ and $\vec{J_{2}}$ the current fluxes (A.m^-2^) across the boundary in either direction.

**Table 1:** Conductivity values for various anatomical structures in the finite element model.

Tissue	Specific conductivity (S.m^-1^)	Assumption and references
Scala tympani, scala vestibule, and scala media	1.5000	Body fluid (Andreuccetti et al., 2017)
Soft tissue surrounding the cochlear ducts	0.25092	Tendon (Andreuccetti et al., 2017)
The cochlear partition, including the organ of Corti	0.017126	Nerve tissue (Andreuccetti et al., 2017)
Basilar and Reissner’s membranes	0.2904	Thorne et al., 2004


Electroporation pulses were modeled as a time-varying electric potential boundary condition on each of the electrode boundaries in contact with the surrounding scala tympani. The voltage waveform outputted by the circuited analysis of the electrode-tissue interface was applied as a Dirichlet boundary condition. To estimate the total resistivity of the tissue for the cases of tandem and alternate electrode configurations, a constant voltage stimulus of 1 V was applied as the Dirichlet boundary condition and the current measured at the boundaries. A Neumann boundary condition (zero flux) was used to model all other boundaries.

The finite element model was solved using an iterative conjugate gradient stationary linear system solver with an algebraic multigrid preconditioner using a successive over-relaxation (SOR) pre-smoother and a backward SOR (SORU) post-smoother algorithm. A backward differentiation formula adaptive time stepping routine was used with a maximum time step of 100 μs. The relative tolerance was set at 10^-2^. The number of degrees of freedom (DOF) was 1,930,229. Quadratic Lagrange basis functions were applied irrespective of the mesh element type. To capture both rapid changes in electric potential as well as steady state values, simulation results were sampled at adaptive intervals ranging between 10 μs and 1 ms.

## Results

### Electrode Impedance Characterization

**FIGURE 2:**
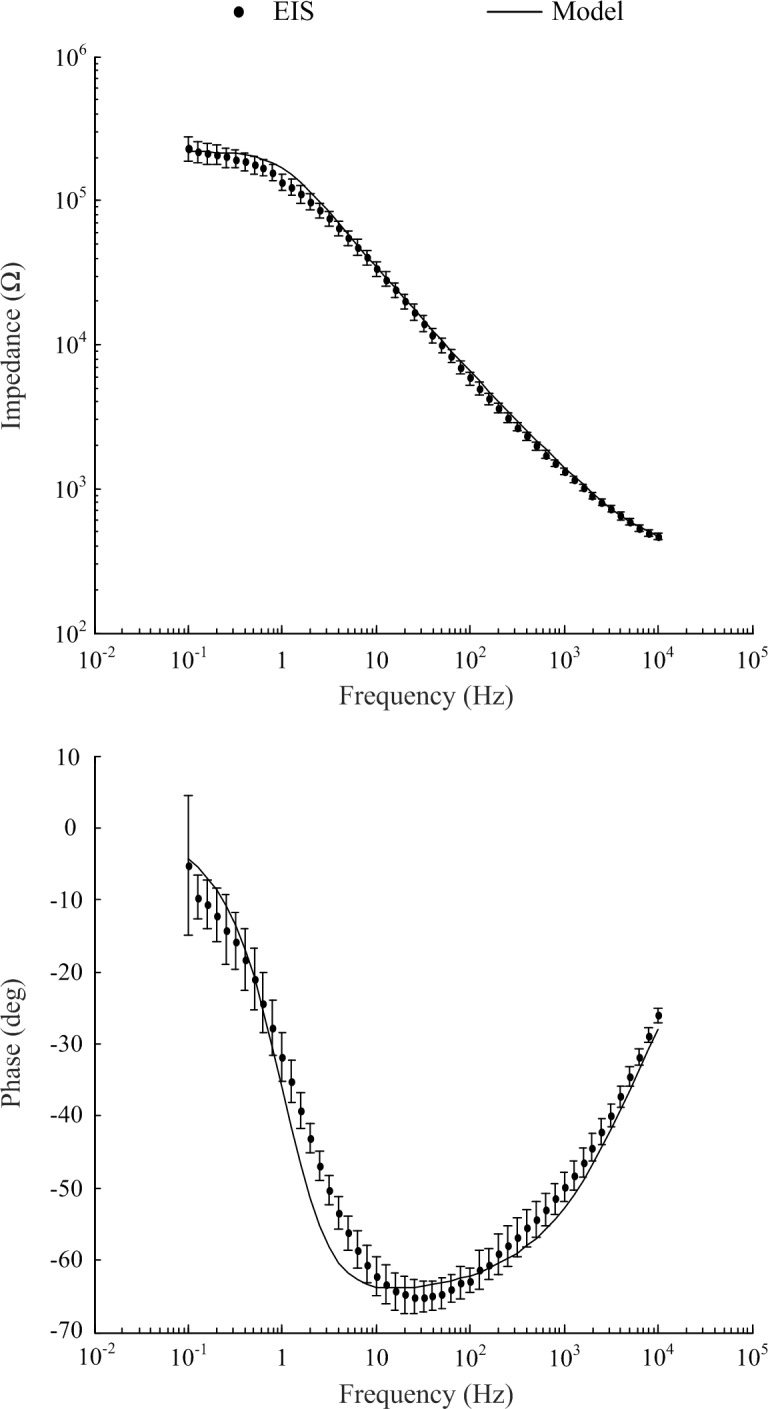
Electrochemical impedance spectrometry characterization of electrodes of the eight channel cochlea array and optimized model to match experimental EIS data (mean ± standard deviation).

Typical of electrodes in electrolytes, impedance was frequency dependent (refer to Figure 2). All eight electrodes displayed a decrease in impedance magnitude as frequency increased and approached a common value at higher frequencies. The standard deviation at 0.1 Hz was 42.3 kΩ and at 10 kHz the standard deviation was 24.17 Ω. The impedance values fell within two standard deviations of the mean. The phase angle peaked at approximately 65° at 12 Hz, with no values falling outside two standard deviations of the mean (refer to Figure 2, bottom panel).

### A Circuit Description of the Electrode-Tissue Interface

**FIGURE 3:**
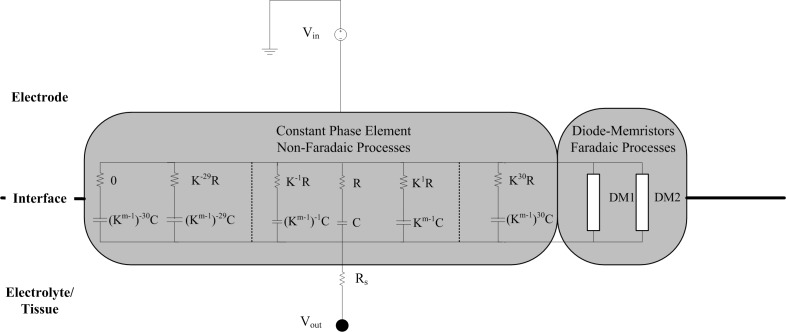
Schematic of the circuit implementation of the electrode-tissue interface. The non-Faradaic processes are modeled as 61 RC branches representing a constant phase element. After selection of the initial R and C values for the middle branch, the resistance and capacitance of all the other branches can be calculated using the m and k parameters. R_s_ is a spreading resistance that represents the contact between the interface and the bulk solution or tissue. Stimulus is provided by a grounded voltage source (V_in_) and delivered, via the interface, to the tissue (V_out_).

The electrode-electrolyte can be considered as a constant phase element (CPE), which we modeled as a network of series resistor-capacitor (RC) branches in parallel to allow for simulation of frequency dependency in the time domain. The time constant of each series-RC branch covers a particular frequency such that the entire network spans the 0.1–10 kHz bandwidth analyzed during EIS measurements. This circuit is coupled with a spreading resistance, representing the contact between the electrode-electrolyte bilayer, and the bulk solution or tissue. Scott and Single (2014) provided a method for determining the necessary parameters of the RC network based on work from Morrison (1959) who provided the mathematical basis of building up the RC network to represent a CPE. Figure 3 shows the topography of the required RC network.

Producing a model with good correlation to the experimental EIS measurements required iterative adjustment of variables. Briefly, to calculate the resistance and capacitance of each branch two parameters are required: the density of RC branches in the network (k), and m the average value of the argument of the CPE. Parameter m is calculated by

$$m=\frac{\pi }{2\theta_{\mathrm{CPE}}}$$

where 𝜃_CPE_ is the phase angle. Model parameters were manually adjusted in the following order to obtain best fit for the impedance magnitude and phase data: number of RC circuits, k, 𝜃_CPE_, and finally R_s_. Optimized model parameters are listed in Table 2 and the values of R and C for each branch are provided in the Supplementary Material.

**Table 2:** Parameters of the constant phase element model of electrodes in the cochlea array.

Constant phase angle (𝜃_CPE_)	65.3°
Density of RC branches (k)	1.18
Number of RC circuits in network	61
Serial resistance (R_s_)	297 Ω


*Parameter values were optimized to reproduce experimental impedance measurements.*

**FIGURE 4:**
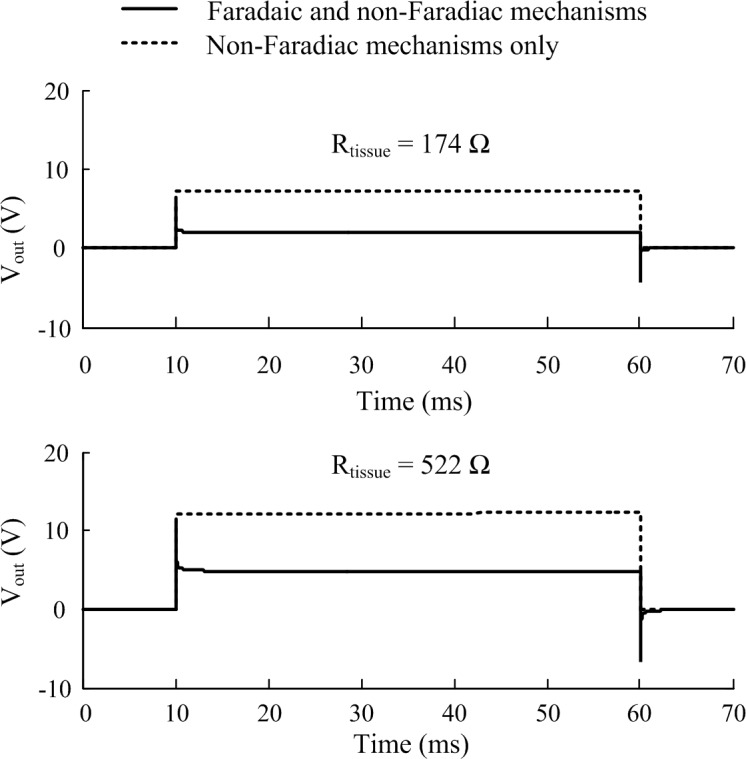
The effect of including Faradaic mechanisms in the electrode-tissue interface model. Two tissue resistance cases are illustrated corresponding to stimulation applied in the alternate and tandem configurations, respectively, in the cochlea. Responses of the interface circuit to a pulse stimulus (20 V, 50 ms, 10 μs rise and fall times) as simulated in LTSpice (1 μs maximum time step size). Faradaic currents were modeled by diode-memristors circuits.

The CPE represents the non-Faradaic mechanisms occurring at the electrode-electrolyte bilayer. To solve the frequency dependent model in the time domain, the 61-branch circuit was implemented as a SPICE model using LTSpice XVII (Analog Devices, United States). The CPE response is characterized by rapid peaks at the onset and termination of voltage pulses and a non-zero steady state of the output potential during the plateau of the pulse (Figure 4), the magnitude of both peaks and steady state are dependent on the resistance of the tissue in contact with the electrode.

A pair of diode-memristor branches was added to phenomenologically represent Faradaic redox reactions occurring at the electrode-electrolyte interface (Scott and Single, 2014) (refer to Supplementary Material for the full circuit diagram). At a stimulus pulse magnitude of 20 V and duration 50 ms, this addition had the effect of increasing the electric potential output across the electrode-tissue interface and masking the CPE peaks at the onset and termination of the stimulus pulse (Figure 4).

**FIGURE 5:**
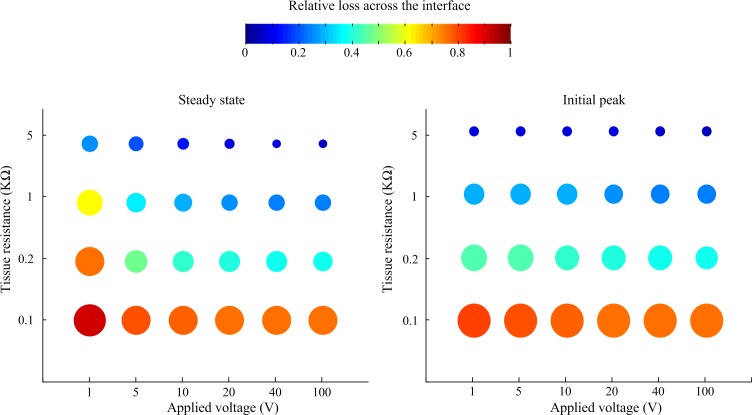
Relative loss of applied voltage across the interface predicted by the LTSpice model. For all cases, the applied rectangular pulse had a width of 50 ms and rise and fall times of 10 μs. The loss was measured at the steady state at the end of the pulse and the initial peak.

A sensitivity study was conducted to systematically investigate the relationship of tissue impedance and applied pulse amplitude on the potential output of the electrode-tissue interface. Using pulses of 50 ms duration and 10 μs rise and fall times, the response of the interface was simulated in LTSpice. The loss in peak and steady-state electric potential across the interface relative to applied voltage is plotted in Figure 5. In general, the relative loss decreased with applied voltage and tissue resistance, although the effect of tissue resistance was more pronounced, especially when considering the initial peak response.

### Finite Element Simulations of Electroporation in the Cochlea

The tissue impedance was predicted *in silico* by applying a constant 1 V stimulus in the alternate and tandem configurations and solving the computational model using a stationary solver. The tissue impedance was predicted to be 174 and 522 Ω for the alternate and tandem configurations compared to 200 and 600 Ω measured experimentally *in vivo*.

**FIGURE 6:**
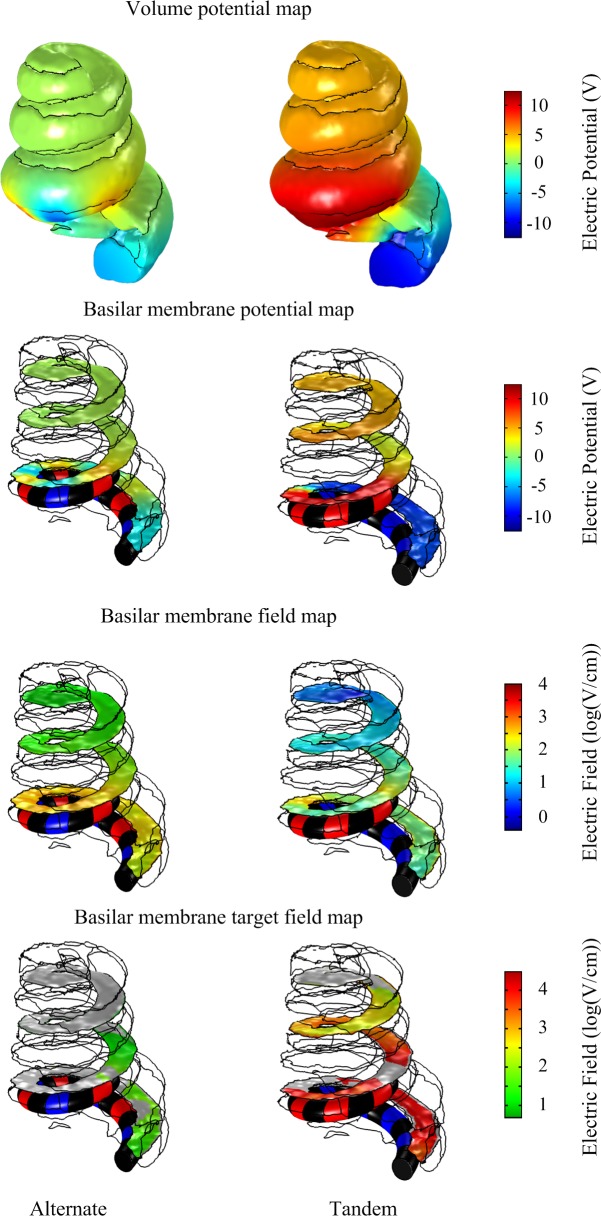
Comparison of electric potential and field maps predicted by a computational finite element model of array-driven electroporation in the guinea pig cochlea. For both alternate and tandem electrode configurations a 20 V, 50 ms pulse was applied with 1 ms rise and fall times. Electric potential and field maps were plotted on the surface representing the basilar membrane.

These *in silico* predicted tissue impedances were used to calculate the voltage drop across the electrode-tissue interface using the circuit model (Figure 4) in response to 20 V, 50 ms monophasic electroporation pulses for both the alternate and tandem configuration (note that the rise and fall times were increased to 1 ms to facilitate numerical convergence in the following step). The predicted voltage waveforms (V_out_ in Figure 4) were subsequently applied in the cochlea FE model to examine the electric field distribution during electroporation. The cochlea experienced electric potentials of ±7 and ±12 V at the cathode/anode electrodes for the alternate and tandem configurations, respectively. The differences between tandem and alternate configurations in terms of both electric potentials and fields are illustrated in Figure 6.

**FIGURE 7:**
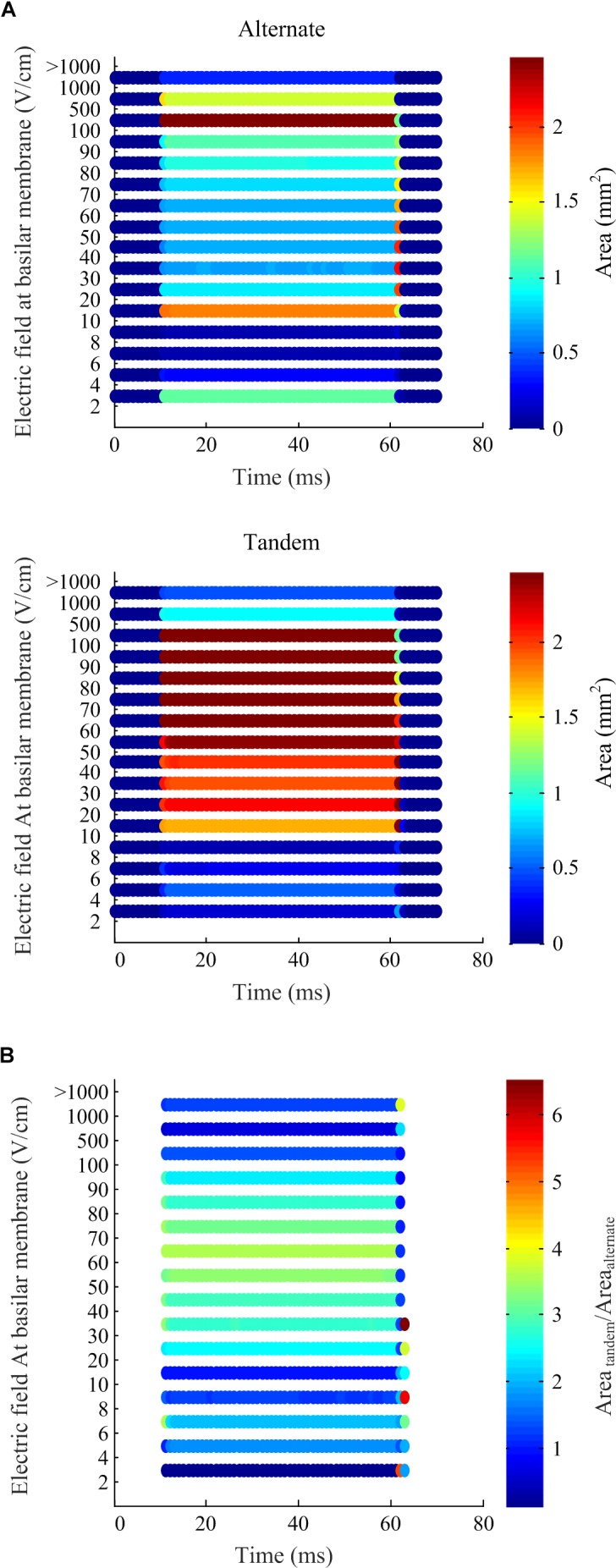
Electric field levels predicted at the basilar membrane by the computational simulation of array-driven electroporation in the guinea pig cochlea. **(A)** The electric field is categorized into different levels and the area on the basilar membrane subjected fallen within each level is plotted at each sample time point. Two electrode configurations are considered: alternate and tandem, and in both cases a single 20 V, 50 ms pulse was applied with 1 ms rise and fall times. **(B)** A comparison of the areas at each electric field level obtained using the tandem electroporation configuration relative to the alternate configuration.

To quantify differences in the electric fields at the target tissue, namely the basilar membrane, we grouped the electric fields at various levels of interest and calculated the areas of target tissue subject to electric field strengths over each range (Figure 7). It is worth noting the spread of areas subject to the electric field within the target range beyond the insertion point of the electrode array. The area of the basilar membrane subjected to electric fields in the 20–100 V.cm^-1^ during array-driven electroporation using the tandem configuration was about three to fourfold that predicted for the alternate configuration (Figure 7). In the 4–8 V.cm^-1^ range an approximately twofold increase in the area in the tandem configuration compared to alternate can be also noted. Also noteworthy is that the alternate configuration generated a large area of target tissue subject to electric fields in the much higher 500–1000 V.cm^-1^ range.

### *In vivo* Gene Electrotransfer

**FIGURE 8:**
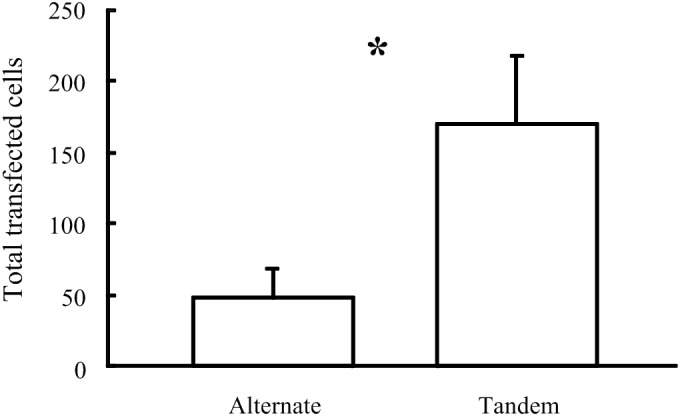
Comparison of the number of cells transfected *in vivo* in the Guinea pig cochlea following electroporation using 40 V pulses delivered by two electrode configurations (^∗^*p* = 0.035).

As described by Pinyon et al. (2014), using five × 50 ms constant-voltage pulses of 20 V in the tandem array configuration achieved much greater DNA electrotransfer efficiency than equivalent pulses using the alternating electrode configuration. Five × 50 ms pulses of 20 V were the most efficient electroporation parameters tested using the tandem array, while with the alternating array configuration, increasing pulse number to 20 and voltage to 40 V improved electrotransfer efficiency. However, even using these increased parameters with the alternating configuration, the mean number of transfected cells was still greater using the tandem configuration (mean = 169.1 ± 47.9, *n* = 7) compared to the alternating configuration (mean = 47.4 ± 21.5, *n* = 5), One-tailed *P*-value = 0.035 (Figure 8).

## Discussion

In contrast to chronic electrical stimulation in neural prostheses where cell or tissue excitability can be related to charge injection and strength-duration curves, hypotheses to explain the novel acute application of bionic array based electric field focusing underlying electroporation based gene delivery (Browne et al., 2016; Housley et al., 2016; Abed et al., 2018) are less established; naked plasmid DNA electrotransfer appears less dependent on membrane pore formation and resealing, than processes of endocytosis (Escoffre et al., 2011). However, both modalities share the premise that efficacy is tied to the strength of the electric field at the target cell or tissue. Therefore it is imperative to be able to map the electric field at target sites *in situ*. For the consideration here of the DNA electrotransfer application, electric fields have been mapped in saline baths *in vitro* and contributed to understanding the improved efficiency of GFP expression using a tandem as opposed to an alternating electrode configuration in HEK293 cell monolayers (Browne et al., 2016). However, the utilized methodology of measuring electric potentials using a microelectrode limits *in vivo* application, and the computational modeling approach is most informative.

In this study, the computational simulation has predicted the electric fields around the complex target tissues within the guinea pig cochlea. Combining electrochemical impedance characterization and modeling of electrodes and anatomically realistic reconstruction of target geometry we simulated array driven electroporation of the cochlea and predicted the electric fields generated at the basilar membrane for both alternate and tandem electrode array configurations. The model’s predictions were verified by comparing and closely matching *in posteriori* differences quantified by imaging of transfected marker proteins following *in vivo* electroporation during similar stimulation protocols.

Finite element computational modeling of electrical stimulation in the cochlea has been the subject of many investigations for over 30 years (Finley et al., 1987; Finley, 1989; Finley et al., 1990). More recent models based on image reconstructions (Ceresa et al., 2014; Kalkman et al., 2015; Kang et al., 2015; Malherbe et al., 2016; Tachos et al., 2016; Teal and Ni, 2016; Wong et al., 2016; Cakir et al., 2017; Schafer et al., 2018) or simplified geometries (Briaire and Frijns, 2000, 2006; Hanekom, 2001; Rattay et al., 2001; Goldwyn et al., 2010; Saba et al., 2014; Nogueira et al., 2016) of the cochlea have been applied to predict electric potential and field distributions following electric stimulation by electrode arrays of cochlear implants.

**FIGURE 9:**
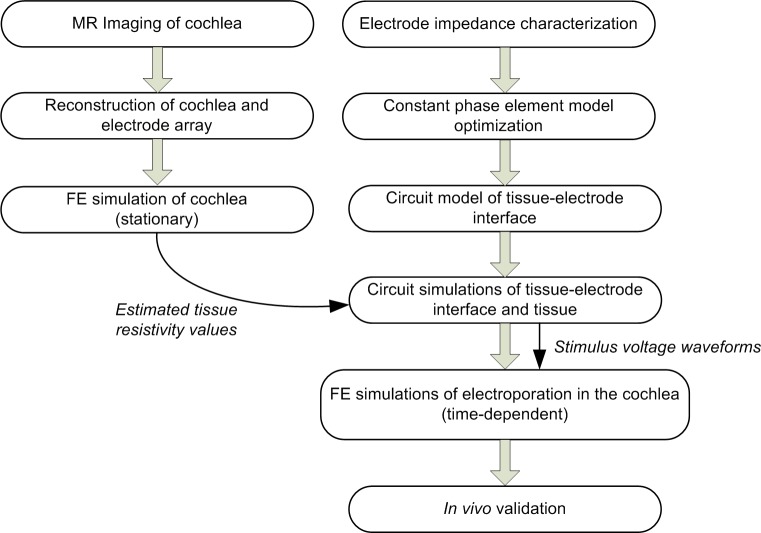
Modeling workflow.

Our 3D finite element electric model is a novel application of computational modeling to study electroporation in the cochlea (Figure 9). The incorporation of time-domain representation of both Faradaic and non-Faradaic mechanisms occurring at the electrode-tissue interface enables simulation of the relatively large-amplitude voltage pulses applied during gene electrotransfer.

The utility of computational modeling to predict generated electric fields during electric stimulation or electroporation pulses is especially demonstrated in cases with complex anatomical and electrode geometries. The helical structure of the cochlea and the winding insertion of the electrode array is a case in point. Our simulations predicted extension of target regions in the cochlea structure distal to the insertion point of the electrode array. This unanticipated finding would have been unattainable by simple analytical calculation of electric fields in tissue between two parallel plate electrodes. Other approaches for estimating electric fields in target tissue or cells include MRI or impedance-based methods (Kranjc et al., 2011). However, these still cannot match the spatial or temporal resolution of computational models.

Impedance of the electrodes, more specifically the electrode-tissue bilayer, is one critical factor in neural interfacing. For stimulating electrodes, an extensive research effort has been targeted toward characterization of impedance with the aim of developing materials and fabrication techniques to reduce the interface impedance and improve long-term biocompatibility. Electrochemical impedance spectrometry has motivated development of mathematical and circuit models of the electrode-electrolyte bilayer that could capture the frequency dependency of interface impedance. This has posed a challenge for developers of computational models of neuroprostheses, whom rely on transient analysis to simulate the responses of tissue to electrical stimulation delivered by the prosthesis. In most 3D finite element cases, the interface has simply been ignored, or replaced by a simple RC circuit that fails to capture frequency-dependency behavior. For small offset potentials, that is non-Faradaic stimulation (Franks et al., 2005; Pham et al., 2010) or the use of recording electrodes (Al Abed et al., 2018), the voltage drop across the interface has been modeled using a cosh function based on the Gouy-Chapman-Stern capacitances (Gouy, 1910; Chapman, 1913; Stern, 1924).

Based on the work by Scott and Single (2014) and Morrison (1959) our electrode-tissue interface model includes formulations for both Faradaic mechanisms, optimized to match experimental EIS measures, and non-Faradaic mechanisms occurring at the bilayer and therefore allows simulation of high voltage pulses typical of electroporation that would most likely drive oxidation and reduction of chemical species at the interface. This is a significant advancement on published computational studies of electroporation that use a voltage boundary condition to model application of pulses to target tissue (e.g., Sel et al., 2007; Corovic et al., 2008; Suzuki et al., 2015). On the other hand, our approach can replicate the voltage drop across the electrode-tissue interface and therefore enables more accurate prediction of the effective electric fields generated in the target tissue.

Our sensitivity analysis predicts that for relatively long voltage pulses (50 ms) the relative loss of applied voltage across the interface decreases with tissue resistance and applied pulse amplitude. In chronically implantable neuroprotheses, development of tissue fibrosis around the implanted electrodes is a major issue reducing the long-term efficacy of implants. Our analysis demonstrates that the impact of fibrosis is complex, for voltage-based stimuli. The voltage drop across the electrode-neural interface will be reduced but at the expense of a higher loss across the extracellular space due to the presence of fibrotic tissue.

It appears that predictions from *in silico* simulations of the cochlea underestimate the tissue impedance for alternate and tandem configurations (174 and 522 Ω for the alternate and tandem compared to 200 and 600 Ω). However, it should be noted that impedance measurements conducted during *in vivo* experiments are measures of total system impedance including the interface impedance. We have shown in circuit simulations that for voltage stimulation the tissue resistance affects the interface impedance and hence the differences in tissue impedance values are expected.

### Gene Electrotransfer in the Cochlea

Cochlear implants can be considered one of the most clinically successful neuroprostheses, with more than 500,000 devices implanted to aid in the restoration of functional hearing in patients. Electroporation facilitated transfection of neurotrophic factors is a methodology that could allow for improving the efficacy of electrical stimulation in cochlear implants (O’Leary et al., 2009; Pinyon et al., 2014, 2019; Browne et al., 2016; Housley et al., 2016; Abed et al., 2018). Even though the model was developed with electroporation as the primary application in this paper, the inclusion of anatomically realistic geometry and EIS-based electrode-electrolyte model makes it well suited to predict electric potentials and fields generated by shorter pulses typically used in cochlear implants for electrical stimulation of the auditory nerve. However, it is notable that current cochlear implant devices cannot produce the sustained current pulses required for CFE due to limitations on rail voltage (∼10 V) and the inductive power supply which is matched to typically low tens of μs pulse durations at kHz frequencies which are effective for auditory nerve fiber excitation, switching dynamically between individual electrodes.

### Model Shortcomings and Limitations

As in all computational simulations studies our model takes a compromise between simplicity of assumptions and accuracy. The model’s description of Faradaic mechanisms at the tissue-electrode interface is purely phenomenological, with no details included for the electrochemical reactions per chemical species at the interface. If knowledge of changes in concentration of ions is required, say for example to predict changes in pH associated with exceeding the water window, an alternative approach that is more focused on the electrochemistry is required. For simplicity we approximated likely tissue damage by identifying target regions subject to high electric field intensities. Based on this we postulate that the alternate configuration yields larger areas with higher electric fields, potentially leading to a higher percentage of cell death.

Another limitation is that the parameters of the diode-memristor circuit representing the Faradaic mechanism at the electrode-tissue interface are taken from Scott and Single (2014) who also used a platinum electrode array in their study albeit of a different size and structure. Future work could include optimizing these parameters for our particular experiments and electrode array.

## Ethics Statement

This study was carried out in accordance with the NSW Animal Research Act 1985, NSW Animal Research Regulations 2010, and Australian Code for the Care and Use of Animals for Scientific Purposes 8th Edition 2013, and were approved by the University of New South Wales Animal Care and Ethics Committee (ACEC approval number 10/81A).

## Author Contributions

FC and AA conducted the EIS measurements. JP and GH conducted the *in vivo* electroporation experiments. GC conducted the MR imaging of the cochlea. FC developed the constant phase element model. AA developed the full electrode–tissue interface LTSpice model. EF segmented and reconstructed the cochlea geometry as well as the electrode array, and generated the 3D mesh. AA and EF developed the computational model. AA drafted the manuscript. AA, JP, GH, and NL designed and coordinated the study. All authors reviewed the manuscript.

## Conflict of Interest Statement

Patents filings associated with this research are managed via New South Innovations, the commercialization arm of University of New South Wales. The authors declare that the research was conducted in the absence of any commercial or financial relationships that could be construed as a potential conflict of interest.
