# Supplementary figures and images for: Computational Simulation Expands Understanding of Electrotransfer-Based Gene Augmentation for Enhancement of Neural Interfaces
Source: Front Neurosci. 2019 Aug 6;13:691. doi: 10.3389/fnins.2019.00691 (PMC6691069; doi:10.3389/fnins.2019.00691)

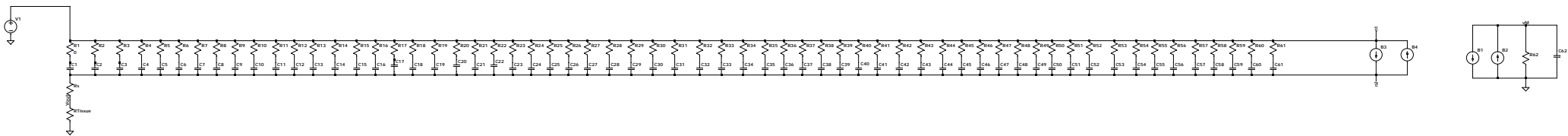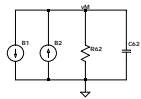

Supplement: Supplementary file 1 [file Data_Sheet_1.PDF]
