# Supplementary material for: Computational Simulation Expands Understanding of Electrotransfer-Based Gene Augmentation for Enhancement of Neural Interfaces
Source: Front Neurosci. 2019 Aug 6;13:691. doi: 10.3389/fnins.2019.00691 (PMC6691069; doi:10.3389/fnins.2019.00691)
Supplement: Supplementary file 2 [file Data_Sheet_2.pdf]

Computational Simulation Expands Understanding of Electrotransfer - based Gene Augmentation for Enhancement of Neural Interfaces

Amr Al Abed[1], Jeremy L. Pinyon[2], Evelyn Foster[1], Frederik Crous[1], Gary J. Cowin[3], Gary D. Housley[2], Nigel H. Lovell[1]

Supplementary Material

1 Constant phase element circuit

A complete circuit diagram is provided in "CPE_61RCbranches_MemristorDiode_VoltageStim.pdf"

The resistor and capacitor values for each branch of the constant phase element circuit:

RC Branch Number	R (Ω)	C (F)
1	0	5.630991019191882e-09
2	82.30416024204867	9.144057179281526e-10
3	97.1189090856174	9.734250849680828e-10
4	114.6003127210285	1.03625379573531e-09
5	135.2283690108137	1.103137720362987e-09
6	159.5694754327601	1.174338598416563e-09
7	188.2919810106569	1.250135063169805e-09
8	222.1845375925752	1.330823731991649e-09
9	262.1777543592387	1.416720367110937e-09
10	309.3697501439016	1.508161111301509e-09
11	365.0563051698039	1.605503803323309e-09
12	430.7664401003686	1.709129378267261e-09
13	508.3043993184349	1.819443358283962e-09
14	599.7991911957531	1.936877439529899e-09
15	707.7630456109887	2.061891181541471e-09
16	835.1603938209667	2.194973805647888e-09
17	985.4892647087406	2.336646109460783e-09
18	1162.877332356314	2.487462504932543e-09
19	1372.19525218045	2.648013187959017e-0
20	1619.190397572931	2.818926448016965e-09
21	1910.644669136059	3.000871126874663e-09
22	2254.560709580549	3.194559235997424e-09
23	2660.381637305048	3.400748742890845e-09
24	3139.250332019956	3.620246537285682e-09
25	3704.315391783548	3.853911588772037e-09
26	4371.092162304587	4.102658308239784e-09
27	5157.888751519412	4.367460126279649e-09
28	6086.308726792906	4.649353302548493e-09
29	7181.844297615628	4.9494409810061e-09
30	8474.576271186441	5.26889750689303e-09
31	10000	5.608973022343303e-09

32	11800	5.970998358616142e-09
33	13924	6.356390244091727e-09
34	16430.32	6.766656848411626e-09
35	19387.77759999999	7.20340368446e-09
36	22877.577568	7.668339891282074e-09
37	26995.54153023999	8.16328492252703e-09
38	31854.73900568319	8.69017566658951e-09
39	37588.59202670616	9.251074026313257e-09
40	44354.53859151327	9.848174987918846e-09
41	52338.35553798566	1.048381521073197e-08
42	61759.25953482307	1.116048217132682e-08
43	72875.92625109122	1.18808238978687e-08
44	85993.59297628762	1.264765933274968e-08
45	101472.4397120194	1.346398936406976e-08
46	119737.4788601829	1.433300856913359e-08
47	141290.2250550158	1.525811771591895e-08
48	166722.4655649187	1.624293707143947e-08
49	196732.509366604	1.729132056908192e-08
50	232144.3610525927	1.840737089035947e-08
51	273930.3460420594	1.959545552010105e-08
52	323237.80832963	2.086022383790628e-08
53	381420.6138289634	2.220662531275055e-08
54	450076.3243181768	2.363992887194199e-08
55	531090.0626954485	2.51657435202277e-08
56	626686.2739806293	2.679004028973868e-08
57	739489.8032971425	2.85191756066712e-08
58	872597.967890628	3.035991616614636e-08
59	1029665.602110941	3.231946541259157e-08
60	1215005.410490911	3.440549172927079e-08
61	218700.9738883638	3.662615844727863e-08

Equations for the arbitrary current sources of the diode-memristor:

B1: `I=500e-12*(1-v(vM))*exp(v(n1,n2)/(2.0*0.026))`
B2: `I=500e-12*(1+v(vM))*exp(v(n2,n1)/(2.0*0.026))`
B3: `I=500e-12*(1-v(vM))*exp(v(n1,n2)/(2.0*0.026))`
B4: I=`500e-12*(1+v(vM))*exp(v(n2,n1)/(2.0*0.026))`

Diode-memristor capacitance and resistance values:

R62: 15kΩ
C62: 200µF

2 Finite Element Mesh Convergence Analysis

Mesh convergence was investigated by comparing the electric field values in the basilar membrane 40 ms following the onset of the 20V 50 ms pulse stimulus. The standard mesh has a maximum element size (defined as the longest edge in a triangle or tetrahedron) of 50 µm. Two additional meshes were tested with maximum element sizes of 75 µm and 100 µm, respectively.

The electric field values were binned into levels. The area of the basilar membrane subjected to an electric field at each level was compared between meshes, and the results are tabulated below.

Tandem Configuration

	Area (mm^2)			Relative difference (%)		
Mesh	Standard Mesh	Coarse Mesh	Coarser Mesh	Standard Mesh	Coarse Mesh	Coarser Mesh
Maximum mesh element size (μm)	50	75	100	100	150	200
*E < 2	0.03653	0.036261	0.03355	100	99	92
2 ≤ E < 4	0.16248	0.14672	0.13822	100	90	85
4 ≤ E < 6	0.52584	0.49011	0.48606	100	93	92
6 ≤ E < 8	0.23396	0.25591	0.25666	100	109	110
8 ≤ E < 10	0.098707	0.10253	0.10448	100	104	106
10 ≤ E < 20	1.7176	1.6872	1.6796	100	98	98
20 ≤ E < 30	2.1507	2.1458	2.1401	100	100	100
30 ≤ E < 40	1.9635	2.0106	2.014	100	102	103
40 ≤ E < 50	2.0405	2.0778	2.0698	100	102	101
50 ≤ E < 60	2.4088	2.4686	2.458	100	102	102
60 ≤ E < 70	2.6186	2.6061	2.589	100	100	99
70 ≤ E < 80	2.655	2.636	2.6026	100	99	98
80 ≤ E < 90	2.6581	2.6099	2.5791	100	98	97
90 ≤ E < 100	2.6696	2.6074	2.5804	100	98	97
100 ≤ E < 500	3.4629	3.5228	3.5378	100	102	102
500 ≤ E < 1000	0.91983	1.0204	1.0557	100	111	115
E ≥ 1000	0.46781	0.53319	0.53796	100	114	115

*The electric field (E) values are in units of V/cm.

Alternate Configuration

	Area (mm^2)			Relative difference (%)		
Mesh	Standard Mesh	Coarse Mesh	Coarser Mesh	Standard Mesh	Coarse Mesh	Coarser Mesh
Maximum mesh element size (um)	50	75	100	100	150	200
E* < 2	0.94491	0.93846	0.93846	100	99	99
2 ≤ E < 4	1.1426	1.1349	1.1349	100	99	99
4 ≤ E < 6	0.29964	0.30749	0.30749	100	103	103
6 ≤ E < 8	0.11069	0.10759	0.10759	100	97	97
8 ≤ E < 10	0.080285	0.081966	0.081966	100	102	102
10 ≤ E < 20	1.8488	1.8572	1.8572	100	100	100
20 ≤ E < 30	0.86385	0.87608	0.87608	100	101	101
30 ≤ E < 40	0.69427	0.70008	0.70008	100	101	101

40 ≤ E < 50	0.71277	0.72513	0.72513	100	102	102
50 ≤ E < 60	0.72317	0.72721	0.72721	100	101	101
60 ≤ E < 70	0.72651	0.72409	0.72409	100	100	100
70 ≤ E < 80	0.83794	0.83243	0.83243	100	99	99
80 ≤ E < 90	0.96364	0.95513	0.95513	100	99	99
90 ≤ E < 100	1.112	1.0969	1.0969	100	99	99
100 ≤ E < 500	2.465	2.4636	2.4636	100	100	100
500 ≤ E < 1000	1.4239	1.4356	1.4356	100	101	101
E ≥ 1000	0.37884	0.38803	0.38803	100	102	102

*The electric field (E) values are in units of V/cm.
